# Effect of virtual reality aggression prevention training for forensic psychiatric patients (VRAPT): study protocol of a multi-center RCT

**DOI:** 10.1186/s12888-018-1830-8

**Published:** 2018-08-06

**Authors:** Stéphanie Klein Tuente, Stefan Bogaerts, Sarah van IJzendoorn, Wim Veling

**Affiliations:** 1 Department of Psychoses, University Medical Center Groningen, Hanzeplein 1, 9713 GZ Groningen, The Netherlands; 2 Forensic Psychiatric Center (FPC) Dr. S. van Mesdag, Helperlinie 2, 9722 AZ Groningen, The Netherlands; 3 Department of Developmental Psychology, Tilburg University, Prof Cobbenhagenlaan 225, PO Box 90153, 5000 LE Tilburg, The Netherlands; 4 Fivoor, Fivoor Science & Treatment Innovation, Kijvelandsekade 1, 3172 AB Poortugaal, The Netherlands

**Keywords:** Aggression, Aggressive behavior, Virtual reality, Forensic psychiatry, SIP model

## Abstract

**Background:**

Many patients residing in forensic psychiatric centers have difficulties regulating their aggression in an adequate manner. Therefore, they are frequently involved in conflicts. Evidenced-based aggression therapies in forensic psychiatry are scarce, and due to the highly secured environment, it is hard to practice real-life provocations. We have developed a Virtual Reality aggression prevention training (VRAPT), providing safe virtual environments, in which patients can practice controlling their aggressive behaviors in an adequate way. The main objective of this study is to examine whether VRAPT is effective in reducing aggression among forensic psychiatric inpatients.

**Methods:**

Four forensic psychiatric centers in the Netherlands are participating in this study. Participants will be randomly assigned to either VRAPT or a waiting list. The two groups will be compared at several different time points: baseline (12 weeks before intervention), pre-intervention, post-intervention and at 12 weeks follow-up. After follow-up measurements are completed, participants from the waiting list will also receive VRAPT. The primary outcome is level of aggressive behavior, consisting of staff-reported and self-reported measures. Secondary outcomes are self-report questionnaires on e.g., anger, impulsivity and aggression.

**Discussion:**

To the best of our knowledge this is the first study to examine the effectiveness of a VR aggression prevention training in forensic psychiatric centers. Further details on the methodological issues are discussed in this paper.

**Trial registration:**

Dutch Trial Register (NTR, TC = 6340). Retrospectively registered 14–04-2017.

## Background

One of the most important issues in working with forensic patients residing in a closed institution is preventing and managing aggressive behavior directed towards staff and other patients [1]. This aggressive behavior not only causes harm to staff and fellow patients, but also has consequences for the treatment progress and the living environment, resulting in longer periods of mandatory treatment. A meta-analysis by Papadopoulos et al. showed that almost 40% of all incidents were preceded by staff-patient interactions. Examples of staff-patient interactions were most frequently denying a patient’s request, limiting a patient’s freedom or some other restriction [2]. The occurrence of incidents in forensic psychiatric centers (FPCs) is not surprising, as forensic patients often have antisocial (or other) personality disorders, lack of impulse control, a high degree of impulsivity and/or a lack of empathy, all factors that are related to aggression [3, 4]. Accordingly, a majority of patients staying in FPCs are admitted under the judicial measure TBS-order (in Dutch: ter-beschikking-stelling: this translates as ‘detained under hospital order’). ‘Detained under hospital order’ means that the court has established a relation between the offense committed and a psychiatric disorder (e.g., [5]). This order applies to serious aggressive offenders having severe psychopathology and who are considered to be at high risk for re-offending.

### Aggression

Aggression is frequently described as having two dimensions [6]. Both dimensions are anchored in the General Aggression Model (GAM; [7]). The GAM is an overarching framework that describes how disruption in cognitive and social processes can lead to aggression. The first dimension, form, concerns the way aggression is expressed: overt (e.g., physical force) or covert (e.g., deliberate manipulation within a relationship). The second dimension, function, relates to the intent of the aggressive behavior [6]. Reactive aggression is an impulsive and under-controlled outburst of anger as a reaction to a perceived or actual threat, provocation or frustration. Proactive aggression refers to a planned or premeditated, controlled display of aggression to achieve personal goals for instance money or power [8, 9]. Statistical methods such as factor analysis can distinguish reactive and proactive aggression. Despite this classification, the concept and measurement of aggression remains the object of debate [9, 10]. Although individuals can engage in both types of aggression, proactive aggression is more often associated with an antisocial and psychopathic personality and with less physiological arousal. Reactive aggression is more commonly related to problems in social information processing; impulsive, anxious or aggressive personality types; and high physiological arousal [11, 12].

With regard to the severity and the unpredictable nature of reactive aggression, it is argued that reactive aggression increases the risk of victimization of staff and fellow patients [13]. Therefore, it is important that effective and early interventions focusing on reactive aggression should be top priority for forensic facilities. A systematic review by Ross et al. showed a significant reduction of physically aggressive behavior following an intervention, thereby demonstrating tentative support for the hypothesis that interventions have a positive effect on the reduction of aggressive behavior [14]. Ross et al. discussed research on psychotherapeutic interventions (e.g., Cognitive Behavioral Therapy) for aggressive and violent behavior in forensic populations. Nevertheless, quality assessments of studies included in this review revealed that there were several methodological flaws; overall, most studies were of average quality and none of them included a power analysis [14].

### Virtual reality in forensic psychiatry

Recently, a few studies have examined the use of Virtual Reality (VR) with forensic populations (e.g., [15]), however none of these studies concerned interventions for aggression regulation. Given the positive results from intervention research in other psychiatric disorders, such as social anxiety and PTSD (for a review see [16]), we suggest that VR could also be used for gradual exposure of forensic patients to controlled, provocative virtual social situations. VR exposure can be used to elicit psychological, physiological and behavioral defensive or aggressive responses, to practice new behavior and to test if patients are increasingly able to control their own and others’ aggression.

A Virtual Reality Aggression Prevention Training (VRAPT) was designed to create a computer based simulation that shows a virtual environment in which patients are confronted with behaviors and experiences of virtual characters in social situations. VRAPT is based on the Social Information Processing (SIP) model [17]. The rationale of the SIP model is the concept that severe reactive aggression problems are due to problems in social information processing. In VRAPT, the VR components follow the different steps of the SIP model (i.e., encoding, interpretation, goal clarification, response generation, response selection and enactment). These steps as applied in the VRAPT are explained in more detail under heading: “Virtual Reality aggression prevention training (VRAPT)”. Furthermore, the VR environments include visual and auditory data and are viewed through stereoscopic glasses. The mechanism of VRAPT is that the patient can engage and practice in de-escalating behavior in interaction with the virtual characters. Practicing allows the patient to learn to recognize imminent aggression of others or himself and to gain control over aggressive impulses. We hypothesize that VRAPT will contribute significantly to the reduction of aggression. Thus leading to reduced aggression both inside and outside FPCs.

### Research aims

The main objective of this study is to investigate the effectiveness of VRAPT in the reduction of reactive aggression in forensic psychiatric inpatients in four Dutch forensic psychiatric centers (FPCs). The primary outcome measure is the level of aggressive behavior, measured by staff-report and a self-report questionnaire. It is expected that both staff-observed and self-reported aggressive behavior will decrease after completion of VRAPT compared to being on the waiting list, and that differences will be maintained after a 12-week follow-up period. Secondary outcome measures are participant’s self-reported rates of anger, impulsivity, hostility, physiological arousal, and coping. As all these different components are contributing to the way a patient reacts to provocation of others.

## Design/methods

### Design

The study is a single blind randomized controlled trial (RCT) with two conditions: (I) the VRAPT condition, in which participants receive the VR training in addition to treatment as usual (TAU) and (II) the waiting list. Participants on the waiting list receive TAU only and are placed on a waiting list to receive VRAPT after the follow-up measurements have been completed. Participants in both conditions are not allowed to participate in any other therapies or trainings specifically focusing on aggression regulation during the research period. The two conditions are compared at four different periods in time. First, a period of 12 weeks staff observations before intervention (baseline), pre-intervention (T1), post-intervention (T2), and after 12 weeks of staff observations a follow-up (T3). The waiting list is offered VRAPT after the end of T3.

### Participants

All patients exhibiting reactive aggression while residing in the participating FPCs, and are considered eligible after checking the inclusion and exclusion criteria, will be asked to participate (see sample size calculation). The four Dutch FPCs taking part are: FPC Dr. S. van Mesdag, located in Groningen; FPC de Kijvelanden, located in Poortugaal; FPC Pompestichting, located in Nijmegen; FPC de Rooyse Wissel, located in Oostrum. After being informed with an information letter, written informed consent is obtained from each participant.

### Inclusion criteria

Participants are forensic psychiatric patients between age 18–65 referred by their treatment supervisor and/or clinical team for aggression training on the basis of pre-admission history of aggression and current problems with reactive aggression. All DSM-5 diagnoses are included and there are no restrictions with regard to (history of) substance use disorders.

### Exclusion criteria

Exclusion criteria are inability to speak and read Dutch, epilepsy, or mental retardation meaning an IQ below 70. In case the documented IQ is below 70, but the treatment supervisors indicate that the IQ measurement is not representative for the current state of intellectual functioning, these patients can still be included as participants.

### Power and sample size calculation

There is no previous research using VR aggression regulation training in patient forensic populations based on which an effect size could be estimated. Conventional aggression regulation therapy in such settings has an effect size of around 0.3 [18]. An effect size of 0.5 is considered as moderate. Using this effect size with a β-power of .80, alpha of .05 and an independent two-sided t-test to evaluate the main outcome, 64 subjects are required in each condition, total *N* = 128.

### Materials

An overview of the materials of the study is displayed in Table 1.

**Table 1:** Overview of assessments

Instrument	12-week observation (baseline)	Pre-intervention	During intervention / waiting list	Post-intervention	12-week observation	Follow-up
SDAS	X		X		X	
AVL		X		X		X
CTQ-SF		X				
NAS-PI		X		X		X
STAXI-2		X		X		X
BIS-11		X		X		X
BDHI-D		X		X		X
RPQ		X		X		X
HIBT		X		X		X
IPQ				X		
Interview (VRAPT only)						X

*SDAS* Social Dysfunction and Aggression Scale *AVL* Aggression Questionnaire *CTQ-SF* Childhood Trauma Questionnaire – Short Form *NAS-PI* Novaco Anger Scale and Provocation Inventory *STAXI-2* State-Trait Anger Expression Inventory-2 *BIS-11* Barratt Impulsiveness Scale *BDHI-D* Buss-Durkee Hostility Inventory-Dutch *RPQ* Reactive Proactive Questionnaire *HIBT* Hostile Intepretation Bias Task *IPQ* I-Group Presence Questionnaire

### Virtual reality aggression prevention training (VRAPT)

VRAPT consists of 16-biweekly individual training sessions. The VR training protocol is primarily based on the theory of Social Information Processing (SIP; [17]). The SIP model is associated with the General Aggression Model (GAM; [7]. The GAM predicts that aggressive reactions are often both reactive and proactive. As described earlier, VRAPT only covers reactive aggression and therefore we focus on the SIP model.

**Fig. 1:**
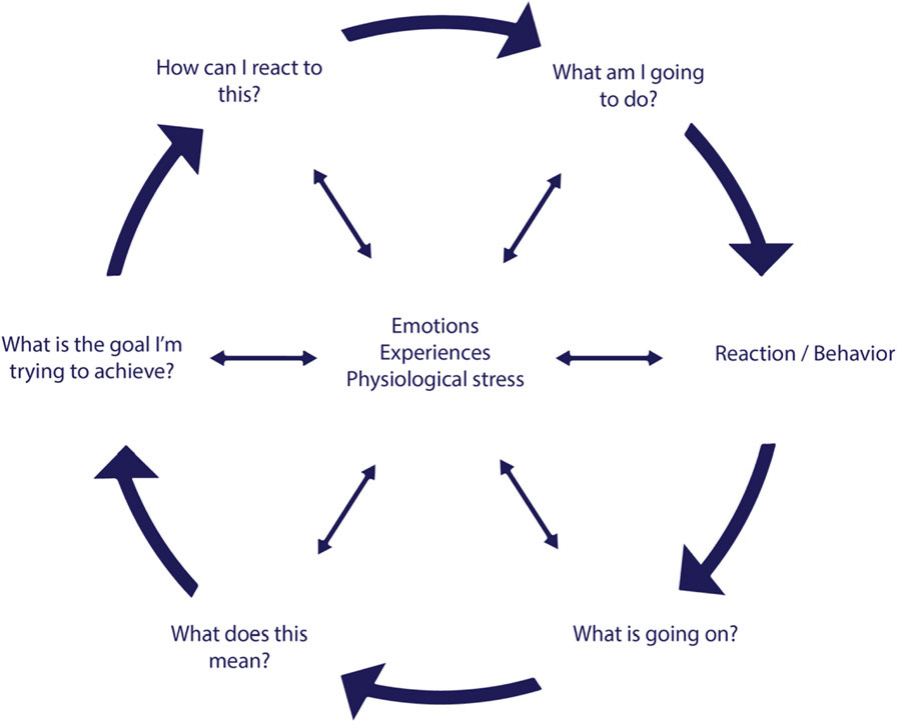
Adapted Social Information Processing model

It is stated that severe reactive aggression problems are caused and remained because of disturbed information processing [17]. Social information processing is a fast and automatic process, involving six different steps that are showed in Fig. 1. This is a simplified version of the original SIP model by Crick and Dodge, so it can also be used as a theoretical model for participants. We transformed the six steps of the model into six questions that can be asked to the participants in different sessions. First encoding: what is going on? Second interpretation: what does this mean? Third selecting a goal: what is the goal I’m trying to achieve in this situation? Fourth generating responses: how can I react to this? Fifth evaluating responses: what am I going to do? And sixth enacting responses: reaction/behavior. These six steps do not only follow each other, but can also influence each other. The center of the circle, including emotions, previous experiences, and physiological stress, all influence the different steps of the SIP model separately.

**Fig. 2:**
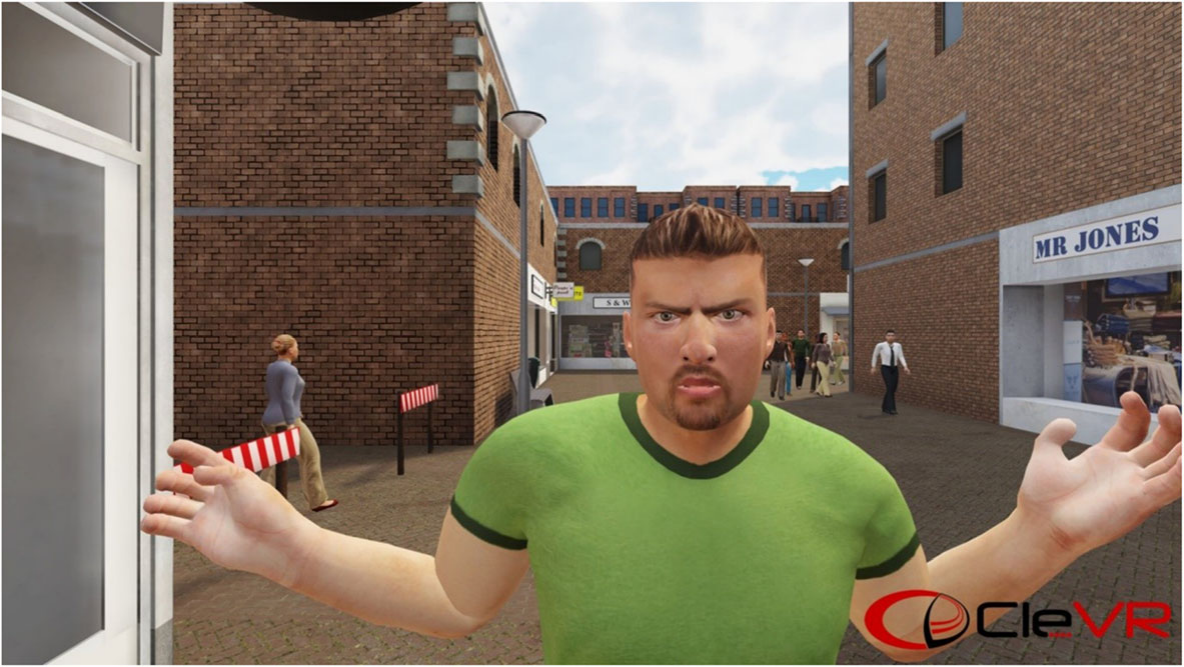
Impression of the virtual environment

In the interactive three-dimensional virtual environment, participants have the opportunity to practice new behavior with virtual characters and learning to cope with their own aggressive behavior in an adequate manner. An example of the virtual environment is displayed in Fig. 2. Each step of the SIP model is first practiced separately in VRAPT. Examples include an exercise on recognition of others’ facial emotions (i.e., what is going on?); rating the level of aggression of virtual character’s behaviors (i.e., what does this mean?); reacting in a sub-assertive manner while being provoked (i.e., how can I react to this?). In the last part of the VRAPT the different exercises will be integrated in more challenging interactive virtual role-plays. Different interactive provocative social scenarios were designed during an iterative process with software engineers, VR experts, clinicians, and researchers. The primary focus of these provocative social scenarios is teaching participants to cope with their reactive aggression in an adequate manner.

During the VRAPT sessions, participants wear headphones and a head-mounted display while interacting with a virtual character that is controlled by the trainer. The trainer takes the role of the virtual character using a microphone with voice distortion for speech, and also manually controlling facial emotion expression and body movements of the virtual character. This highly dynamic interactive nature of the VR system means VRAPT can be tailored to the specific needs of the participants, and participants have the opportunity to practice with their own learning goals and difficulties. Additionally, real-time galvanic skin response (GSR) and heart rate (HR) will be measured as feedback for participants on their physical arousal during the sessions. Furthermore, trainers will stimulate participants to continue applying novel behavior they have learnt in VR. At all times the trainer is in control of the virtual environment and is able to immediately change and/or stop the virtual environment if necessary.

### Waiting list (control condition)

Participants in both the VRAPT and waiting list condition receive standard treatment including: supportive counseling by treatment staff, medication and psychological treatment only if it is not directly focused on aggression regulation (allowed e.g., schema therapy, Liberman training, and treatment for addiction problems). Because randomization is on individual level, participants in both conditions may be residing on the same ward and therefore both conditions have access to the same treatment facilities.

### Fidelity checks

The VRAPT protocol is designed as guidance for the trainers and to ensure treatment integrity. Each session in the protocol follows the same format: short discussion of previous session, clarification of the step of the SIP model, conversation about the VR assessment, technical guidance and each session ends with an evaluation of the learning goals. Besides, VRAPT-trainers receive 16 h of training in using the VRAPT system and the protocol. The training is provided by the first and third author, respectively the researcher and a highly skilled psychologist. In addition to this training, monthly one-hour group multicenter videoconference supervision serves to guide the trainers throughout the intervention period and is facilitated by one of the two clinical experts; the third and last author. Furthermore, during the intervention, trainers are required to complete session forms, which are checked for completeness by the research assistant afterwards. Finally, at two different moments during the VRAPT sessions, trainers have to send their learning goals to a highly skilled clinician within their own organization for feedback.

### Measurement instruments

#### Primary outcome

##### Level of aggressive behaviour

Primary outcome measure is level of aggressive behaviour. Self-report questionnaires and staff-reports are used to assess the effectiveness of VRAPT, because the perspectives of patients and staff represent distinct but complementary measures of aggressive behaviour.

##### The social dysfunction and aggression scale (SDAS)

Staff is trained and asked to complete the Social Dysfunction and Aggression Scale (SDAS; [19, 20]) weekly for each patient meeting the inclusion criteria and signed informed consent, to document the aggressive state of patients. SDAS data is collected at least 12 weeks before VRAPT starts. The SDAS provides systematic recording of staff observations on a broad range of aggressive behaviour on a weekly basis. In this research, the 9-item version was chosen. Illustrative examples are: *irritability*, e.g., difficulty controlling reactions; *negativism*, e.g., not wanting to cooperate; *directed verbal aggressiveness*, e.g., insulting people personally. All 9 items are scored along a 4-point scale ranging from absent to severely present.

In the current study, the Dutch SDAS manual of Bousardt is used. As described in this manual, for each of the items a general and peak score can be scored [21]. In this way, each SDAS item is scored twice, first on the most severe aggressive behaviour (peak), and second on average level of aggressive behaviour. SDAS scores of participants in the VRAPT condition (intervention group) will be compared with the SDAS scores of participants on the waiting list (control group). Psychometric properties of the Dutch version of the SDAS are moderate inter-rater reliability and good convergent validity in a FPC (Cronbach’s α (11 items) = .82; [20]).

##### Aggression questionnaire (AVL)

In addition, participants are asked to the Dutch version of the Aggression Questionnaire (AVL; [22]) at three different measuring points: pre-intervention (T1), post-intervention (T2), and 12-week follow-up (T3). This questionnaire assesses four sub traits of aggression, i.e., physical aggression, verbal aggression, anger and hostility [23]. The test–retest reliability of the AQ total scores were good and validity showed significant correlations with alternative aggression measurements. A Dutch study showed good internal consistency (Cronbach’s α (total, inpatients) = .83; [24]).

#### Secondary outcome measures

Components of the SIP model are used as secondary outcomes and are assessed with diverse self-report questionnaires.

##### Child trauma questionnaire-short form (CTQ-SF)

The Child Trauma Questionnaire-Short Form (CTQ-SF; [25]) is used to measure childhood neglect and abuse, which are predictive factors for victimization and being a perpetrator in adulthood [26]. Moreover, male prisoners scoring high on the CTQ-SF were significantly more often involved in violent behaviour during their stay in prison [27]. The Dutch version of the CTQ-SF demonstrated adequate internal consistency reliability in a large community sample including amongst other patient groups forensic patients and prisoners (Cronbach’s α ≥ .63; [28]).

##### Barratt impulsiveness scale (BIS-11)

The Barratt Impulsiveness Scale (BIS-11; [29] is a 30-item measurement used to study the role of impulse control [30]. The BIS-11 has three subscales, namely: attentional impulsiveness, non-planning impulsiveness, and motor impulsiveness. A study showed that the BIS-11 is sensitive to different levels of aggression [31]; non-violent offenders scored lower on the BIS-11 than their violent counterparts (Cronbach’s α = .81; [32]).

##### Buss-Durkee hostility inventory-Dutch (BDHI-D)

The Buss-Durkee Hostility Inventory-Dutch (BDHI-D; [33]) is used to measure two factors of aggression: covert or indirect aggression (20 items) and overt or direct aggression (20 items), and is rated on a ‘true’- ‘not true’ dichotomous scale [34]. The combination of verbal and physical aggression represents direct aggression, whereas hostility and anger are the core concepts of indirect aggression (Cronbach’s α (Overt Aggression) = .77, α (Covert Aggression) = .79; [34]).

##### Novaco anger scale and provocation inventory (NAS-PI)

The Novaco Anger Scale and Provocation Inventory (NAS-PI; [35]) is a two-part test of 73 items designed to assess anger as a problem of psychological functioning and physical health and to assess therapeutic change [35]. In Dutch forensic psychiatric patient samples, the internal consistency of the NAS-PI is excellent (Cronbach’s α (NAS, total) ≥ .92, α (PI, inpatients) = .90). Furthermore, in a forensic outpatient sample the test-retest reliability was good [35].

##### State-trait anger expression Inventory-2 (STAXI-2)

The State-Trait Anger Expression Inventory-2 (STAXI-2; [36]) is used to measure the experience, expression, and control of anger. The STAXI-2 is a 57-item measure with scales developed to assess anger on three different scales: 1) state anger scale, this is situational anger; 2) trait anger scale, this is a dispositional characteristic, and 3) anger expression scale, this is the expression of anger [37]. The Dutch version of the STAXI-2 has adequate psychometric properties (Cronbach’s α ≥ .71; [38]).

##### Reactive-proactive questionnaire (RPQ)

Reactive-Proactive Questionnaire (RPQ) is a self-report of aggression that is not restricted to a short period of time. The RPQ is a questionnaire in which the person is asked about the reasons and the type of aggressive behaviour and refers to this behaviour in general [39]. The RPQ has an excellent internal consistency (Cronbach’s α .91; [40]).

##### Hostile interpretation Bias task (HIBT)

Hostile Interpretation Bias Task (HIBT) is a 10-min computer task to measure hostile interpretation of ambiguous facial expressions of emotions [41].

##### I-group presence questionnaire

The Igroup Presence Questionnaire (IPQ) is used to measure sense of presence in the virtual environment. The IPQ consists of 14 items, responses are made on a 7-point Likert scale, and it has demonstrated good psychometric properties [42].

##### Interview

At follow-up, patients in the VRAPT condition will be interviewed by the research assistant to assess their own opinion about VRAPT. Questions such as: “how did VRAPT contribute to your personal aggression management?” will be asked. This will contribute to the secondary outcome measures, as specific questions about VRAPT can be asked in addition to the self-report questionnaires.

### Procedure

**Fig. 3:**
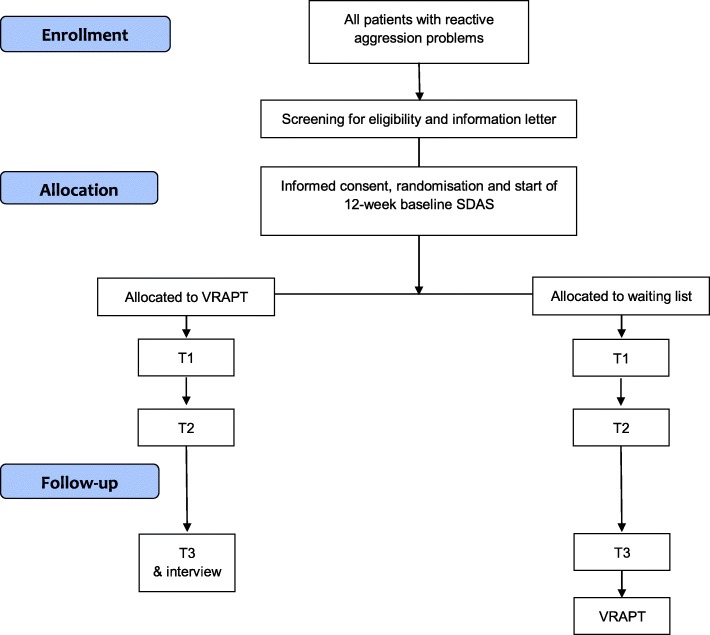
CONSORT Flow Diagram

All patients are screened by their treatment supervisors based on the in- and exclusion criteria. Patients who meet inclusion criteria are invited by an independent research assistant to participate, and receive an information letter (Fig. 3). Patients have 1 week to consider participation, after which written informed consent is obtained. Next, participants are randomly allocated to either VRAPT or waiting list condition and are monitored with the SDAS by staff for aggression on a weekly basis during 12 weeks before start of the intervention until the end of the follow-up. Before the intervention starts, pre-intervention measures are obtained (see Table 1). During 10 out of 16 sessions of VRAPT physiological measures of emotional arousal (i.e., heart rate and galvanic skin response) are measured. VRAPT consists of 16 bi-weekly individual sessions with a maximum duration of 60 min per session. The participants in the control condition will be placed on a waiting list. They will have the same assessments as the VRAPT group, i.e., the 32-weeks SDAS, pre-intervention, post-intervention and follow-up measurements. They will receive VRAPT after the last follow-up measurement. During the study period, for both conditions, treatment as usual will proceed, except for specific anger or aggression management therapy. During the intervention, aggression keeps being monitored with the SDAS by staff working on the ward. After the last session of the intervention, pre-intervention self-report measures are repeated in all participants, guided by assessors who are blinded for treatment allocation. During 12 weeks after end of the intervention, participants keep being monitored for aggressive behavior. Twelve weeks after the last session, self-report measures are repeated again.

### Randomization

Participants will be randomly assigned to one of two conditions: VRAPT or waiting list. Randomization is conducted via a scientific randomization program on the Internet (https://www.randomizer.org), by the research coordinator of the Department of Psychiatry from the University Medical Center Groningen. Twenty-two sets consisting of two unique numbers are made available for each participating FPC. Research assistants are informed about the randomization by e-mail and communicate the condition verbally to the participants and the clinical teams.

### Statistical analysis

Analysis of the data will be conducted according to the intention to treat principle [43]. The result of randomization will be checked by comparing baseline socio-demographic and clinical parameters between VRAPT and the waiting list condition, using univariate analyses (chi-square tests for dichotomous measures and t-tests for continuous measures). Differences in scores between pre-intervention, post-intervention and follow-up assessments and between intervention and control group will be examined for the dependent variables, using multilevel (repeated-measures) random linear regression analysis. Primary outcome measure is peak and general score as measured with the SDAS and self-report aggression measurement of participants. The SDAS and self-report measurement will be compared between VRAPT and waiting list, before, during and after VRAPT or waiting list. Effects of VRAPT will be tested with group x time interaction terms. Risk factors for aggression will be used as covariates in the linear regression models. Significance of fixed effects are assessed by Likelihood Ratio tests. Statistical significance of the regression effects will be tested using T-tests. In all analyses, a *p*-value < .05 will be considered statistically significant.

## Discussion

The main goal of this study is to investigate the effectiveness of VRAPT on reactive aggressive behavior of forensic psychiatric inpatients. This study will be the first to evaluate the effectiveness of a VR intervention targeted at reducing reactive aggressive behavior of forensic patients. The hypothesis of the study is that VRAPT will decrease both self-reported and staff-reported aggressive behavior.

Inpatient aggressive behavior is a predictor for future aggression in society, because inpatient aggression is related to non-compliance, higher treatment dropout, and more personality disorders [44]. Current interventions focusing on the reduction of aggressive behavior have several limitations [18]. First, exposure to provocation in highly secured forensic settings is limited. Thus, it is not possible to practice and train forensic patients to control the anger of others and themselves by provoking them in real life social situations. Second, social skill training to prevent aggression by others is limited, and is usually practiced only in treatment groups and role play. Third, engaging forensic patients in treatment is challenging. Attrition is high, as many of them do not like treatment, and/or find it difficult to apply cognitive therapeutic insights. Fourth, it is difficult to evaluate objectively and reliably to what extent patients have learnt to regulate their aggressive impulses, which makes forensic risk assessment complicated.

Addressing the previously listed limitations, VRAPT has several advantages. First, patients can have the opportunity to practice with difficult behavior and additionally gain tools to cope with aggression, instead of leaving the situation or avoiding angry emotions. Second, VR is an interactive tool that allows trainers to provoke and trigger aggression during the training. Often this is described as a difficult aspect by trainers, however, with VRAPT the trainers feel safer in provoking participants, as the participant faces the virtual character and the trainer’s voice is transformed. This also works the other way around: because the trainer ‘plays’ as the virtual character, it is safer for the participant to express his aggression without worrying about the therapeutic relationship. Third, VRAPT is an experiential, behavioral training, focusing on practicing behavior and not necessarily on gaining cognitive therapeutic insights. Fourth, objective and reliable assessment of aggression in real life situations on treatment wards is difficult. Therefore, in the VRAPT real-time measurements of physiological arousal are included. These measurements help to gain more insight into arousal of the patients. Moreover, not only self-report measurements, but also staff observations are recorded. All these aspects are of specific importance because forensic psychiatric patients are at an increased risk of behaving in an aggressive manner.

From a broader perspective, this study will shed light on the effectiveness of VR interventions in forensic populations. If proven to be effective, VRAPT could be a useful addition to current interventions in forensic psychiatry and be implemented in other forensic psychiatric centers, especially as evidence based interventions to reduce aggressive behavior are scarce [14]. In addition, reducing aggression of forensic patients is of great importance for patients, but also for staff working in forensic psychiatric centers, as many fellow patients and treatment staff are victimized by aggression of forensic patients. Because patients can practice safely with difficult situations that they can expect to be confronted with in the outside world, VRAPT may be an important contribution to the preparation for reintegration into society. Thus VRAPT is expected to lead to improvement of psychological, social and emotional well-being of patients and treatment staff.

### Trial status

Ongoing.

## Abbreviations

AVL: Aggression Questionnaire
BDHI-D: Buss-Durkee Hostility Inventory-Dutch
BIS-11: Barratt Impulsiveness Scale
CTQ-SF: Childhood Trauma Questionnaire – Short Form
DSM: Diagnostic and Statistical Manual of Mental Disorders
FPCs: Forensic Psychiatric Centers
GAM: General Aggression Model
GSR: galvanic skin response
HIBT: Hostile Interpretation Bias Task
HR: heart rate
IPQ: Igroup Presence Questionnaire
IQ: Intelligence quotient
NAS-PI: Novaco Anger Scale – Provocation Inventory
NTR: Dutch Trial Register
NWO: Netherlands Organization for Scientific Research
PTSD: Post-Traumatic Stress Disorder
RCT: Randomized Controlled Trial
RPQ: Reactive-Proactive Questionnaire
SDAS: Social Dysfunction and Aggression Scale
SIP model: Social Information Processing model
STAXI-2: State-Trait Anger Expression Inventory-2
TAU: Treatment As Usual
TBS-order in Dutch: ter-beschikking-stelling: this translates as ‘detained under hospital order’
VR: Virtual Reality
VRAPT: Virtual Reality Aggression Prevention Training
